# The Exocyst Complex Subunit EXO70E1-V From *Haynaldia villosa* Interacts With Wheat Powdery Mildew Resistance Gene CMPG1-V

**DOI:** 10.3389/fpls.2021.652337

**Published:** 2021-07-08

**Authors:** Jia Zhao, Heng Zhang, Xu Zhang, Zongkuan Wang, Ying Niu, Yiming Chen, Li Sun, Haiyan Wang, Xiue Wang, Jin Xiao

**Affiliations:** ^1^State Key Lab of Crop Genetics and Germplasm Enhancement, Cytogenetics Institute, Nanjing Agricultural University/JiangSu Collaborative Innovation Center for Modern Crop Production, Nanjing, China; ^2^The Laboratory of Seed Science and Technology, Guangdong Key Laboratory of Plant Molecular Breeding, Guangdong Laboratory of Lingnan Modern Agriculture, State Key Laboratory for Conservation and Utilization of Subtropical Agro-Bioresources, South China Agricultural University, Guangzhou, China; ^3^State Key Laboratory for Managing Biotic and Chemical Threats to the Quality and Safety of Agro-products, Institute of Virology and Biotechnology, Zhejiang Academy of Agricultural Sciences, Hangzhou, China

**Keywords:** EXO70E1-V, CMPG1-V, *Haynaldia villosa*, interaction protein, powdery mildew

## Abstract

EXO70 belongs to the exocyst complex subunit that plays a critical role in regulating plant cell polarity establishment and defense response. A previous study proved that the E3 ligase CMPG1-V from *Haynaldia villosa*, a diploid wheat relative, positively regulates the resistance to wheat powdery mildew (*Pm*), caused by fungus *Blumeria graminis* f.sp *tritici* (*Bgt*). In this study, a member of EXO70 superfamily named *EXO70E1-V* was isolated from *H. villosa*, and *EXO70E1-V* interacted with CMPG1-V were shown by yeast two-hybrid (Y2H), pull-down assay, bimolecular fluorescence complementation (BiFC) assay, and luciferase complementation imaging (LCI) assay. It is localized in various subcellular organs, i.e., plasma membrane (PM) and endoplasmic reticulum. Co-expression of EXO70E1-V and CMPG1-V showed dot-like structure fluorescence signals that were mainly in PM and nucleus. Expression of *EXO70E1-V* was relatively higher in leaf and was significantly induced by *Bgt* infection and exogenous application of hormones such as salicylic acid. Transient or stable overexpression of *EXO70E1-V* could not enhance/decrease the *Pm* resistance level, suggesting overexpression of EXO70E1-V alone has no impact on *Pm* resistance in wheat.

## Introduction

The exocyst complex is a conserved octameric vesicle–tethering complex with principal roles in mediating secretory vesicle to the target membrane prior to SNARE-mediated fusion (Sabol et al., 2017; Kulich et al., 2018). EXO70, a key subunit of the exocyst complex, serves as a spatial landmark of the exocyst complex at the active exocytosis sites (Wu and Guo, 2015). There are multicopies of EXO70 in land plants (Ma et al., 2016). In coordination with other proteins, different members play a diverse role in the processes of cell polarity establishment, including a deposition of seed coat pectin (Kulich et al., 2010), xylem development and formation of Casparian strip (Li et al., 2013; Kalmbach et al., 2017), vascular bundle differentiation (Tu et al., 2015), pollen tube and trichome cell wall maturation (Fendrych et al., 2010; Synek et al., 2017; Kulich et al., 2018), leaf senescence and legume growth and development (Wang et al., 2016), and transport of PIN auxin carriers to specific parts of the plasma membrane (PM) (Drdova et al., 2013).

The important role of EXO70 subunits in plant defense responses against biotic stresses has been reported. *AtEXO70B2* is a positive regulator in plant–pathogen interaction. It interacts with secreted defense-related protein SNAP33 and negative regulator PUB22 in PAMP-triggered responses, which prevents pathogens from infecting cells by increasing the cell wall thickness (Pecenkova et al., 2011; Stegmann et al., 2012). *AtEXO70B1* is highly homologous with *AtEXO70B2*, which can be recruited to the cytoplasmic membrane by RIN (Sabol et al., 2017). AtEXO70B1 involves in plant defense responses by interacting with RIC7 and PUB18 to regulate the stomatal movement (Hong et al., 2016) or phosphorylated by CDPK5 and interacts with the atypical immune receptor TIR-NBS2 to block the growth of pathogens (Zhao et al., 2015; Liu et al., 2017). Moreover, *AtEXO70E2* is essential for exocyst subunit recruitment to form a new organelle named EXPO. EXPO is a novel spherical double-membrane structure involved in unconventional protein secretion for cytosolic proteins (Wang et al., 2010; Ding et al., 2014; Robinson et al., 2016). OsEXO70E1 interacts with Bph6 to enhance the exocytosis and strengthen the cell wall, thereby hindering the feeding of the planthopper and as a result, improving the resistance (Guo et al., 2018).

Common wheat (*Triticum aestivum* L., 2*n* = 42, AABBDD) is one of the most important food crops, which is the staple food for at least one-third of the population of the world (Shi and Ling, 2018). The release of the draft genome sequence allows us to identify and analyze all the members of EXO70 superfamily (Zhao et al., 2018). In our previous study, a U-box/Arm-type E3 ligase gene *CMPG1-V* was cloned from *Haynaldia villosa*. The functional study demonstrated that CMPG1-V is a positive regulator in powdery mildew (*Pm*) resistance of common wheat (Zhu et al., 2015). However, the regulation mechanism remains unknown.

In this study, a yeast two-hybrid (Y2H) screening was performed by using CMPG1-V as a bait, and a subunit of the exocyst complex was identified and cloned from *H. villosa*, namely *EXO70E1-V*. The interaction between EXO70E1-V and CMPG1-V *in vivo* and *in vitro* was verified by pull down assay, luciferase complementation imaging (LCI) assay, and bimolecular fluorescence complementation (BiFC) assay. *EXO70E1-V* was significantly upregulated in response to *Blumeria graminis* f.sp *tritici* (*Bgt*) inoculation and exogenous salicylic acid (SA) treatments. Nevertheless, the functional analysis suggested that overexpression of EXO70E1-V alone has no impact on *Pm* resistance in wheat.

## Materials and Methods

### Plant Materials

*Haynaldia villosa* (2*n* = 14, VV, accession no. 91C43) was used for cloning and expression analysis; a set of *T. aestivum*–*H. villosa* addition lines (DA1V–DA7V), each contains one pair of chromosomes from *H. villosa* in Chinese Spring background used for chromosome location; wheat cultivar Yangmai158 (moderately susceptible to *Bgt*) was used for the subcellular localization analysis and as receptors for stable transformation; Sumai3 was used for propagation of the freshly mixed races of *Bgt* spores, and all of those materials were developed or preserved by Cytogenetic Institute, Nanjing Agricultural University (CINAU). *Nicotiana benthamiana* plants were grown in a controlled growth room at 24°C/20°C day/night with 12 h/day light and 70% humidity. The 5- to 6-week-old plants were used for *Agrobacterium*-mediated transient expression.

### The Chemical Treatments of Plants

The *H. villosa* seedlings were grown in liquid with constant 14 h light/10 h dark (24°C/18°C, 70% humidity). At the three-leaf stages, the plants were inoculated with *Bgt* and treated with 100 μg/mL insoluble chitin (No. C7170, Sigma-Aldrich, United States) or 0.1 mmol/L flg22. Meanwhile, the treatments with exogenous hormone or signal molecules, including sprayed with 5 mmol/L SA, 0.1 mmol/L methyl jasmonate (MeJA), 0.1 mmol/L ethylene (ET), 0.2 mmol/L abscisic acid (ABA), and 7 mmol/L hydrogen peroxide (H_2_O_2_), respectively, were conducted. All samples were collected after 0, 1, 4, 8, 12, 24, 48 h treatments and rapidly frozen in liquid nitrogen and then stored in ultra-freezer (–80°C) until used.

### Yeast Two-Hybrid Protein–Protein Interaction

The yeast strain AH109 was cultured on YPAD plates that were used to test protein to protein interaction between CMPG1-V and EXO70-V. In brief, the full-length CMPG1-V fused with the DNA-binding domain of GAL4 in pGBKT7, while the EXO70-V cDNA sequence was inserted into a pGADT7 vector harboring an activation domain (AD) (Faheem et al., 2016). The transformation mixtures were plated on SD-Leu-Trp medium incubation at 28°C for 2–3 days, and then the individual clones were spotted onto selection media SD-His/Leu/Trp with X-α-Gal (40 mg/ml). The primers are listed in Supplementary Table 1.

### Firefly LCI and BiFC Assays

For firefly LCI assay, the coding regions of EXO70E1-V (without the stop codon) and CMPG1-V were ligated into the pCAMBIA-NLUC and pCAMBIA-CLUC vectors, respectively. For BiFC assays, the EXO70E1-V and CMPG1-V were fused with the N-terminus or C-terminus of the split-yellow fluorescent protein (YFP), respectively. The primers are listed in Supplementary Table 1. Different recombinant plasmids including NLUC-EXO70E1-V, CLUC-CMPG1-V, YN-EXO70E1-V, and YC-CMPG1-V with the control vector were introduced into *Agrobacterium tumefaciens* strain GV3101. Overnight agrobacterial cultures were resuspended with infiltration buffer (10 mM MgCl_2_, 0.1 mM acetosyringone, and 10 mM MES). Different experimental and control group agrobacterial suspensions were mixed and co-infiltrated into 5- to 6-week-old *N. benthamiana* leaves by using a needleless syringe and then weak light growth. Two days later, for LCI, 1 mmol/L luciferin was infiltrated into the leaves, and the plants were kept in the dark for 10 min. LCI images were captured using a low-light cooled CCD imaging apparatus, as described by Chen et al. (2008). For BiFC, fluorescence was observed by confocal microscopy.

### *In vitro* Pull-Down Assay

The full-length open reading frame (ORF) of EXO70E1-V was individually ligated into *pGEX6p-1* vector for protein expression, and the primers are listed in Supplementary Table 1. The recombinant glutathione *S*-transferase (GST)-EXO70E1-V plasmid and control vector were expressed in the *Escherichia coli* strain BL21 (DE3) and purified by anti-chromatography using glutathione sepharose beads. Generation of the Maltose-Binding Protein (MBP)-CMPG1-V construct and protein purification were performed as described by Zhu et al. (2015). For pull-down assay, the GST-EXO70E1-V and GST alone (control) proteins were incubated with glutathione sepharose beads at 4°C for 2 h with gentle shaking and then incubation with MBP-CMPG1-V and MBP proteins, respectively, for 1 h at room temperature. After incubation, the beads were harvested, washed once with PBS buffer containing 500 mM NaCl, and subsequently washed five times with the same buffer containing 135 mM of NaCl. The bound protein complex retained on the beads was extracted by boiling the beads in 10 μL of 2× SDS-PAGE loading buffer and finally analyzed by western blotting using GST and MBP antibodies.

### Phylogenetic Analysis

The amino acid sequences of *Arabidopsis* EXO70 proteins were downloaded from the Ensembl Plants database^1^. All EXO70 protein sequences were aligned by ClustalW, and a phylogenetic tree was constructed by MEGA6 using the neighbor-joining method with the pairwise deletion option, Poisson correction, and bootstrap analysis with 1,000 replicates (Tamura et al., 2013).

### RNA Extraction, cDNA Synthesis, and Gene Expression Profiling

Total RNA was extracted by using the Trizol Reagent Kit (Invitrogen, Carlsbad, CA, United States) and analyzed by gel electrophoresis. The first-strand cDNA was synthesized with random oligonucleotides using the HiScript^®^ II Reverse Transcriptase system (Vazyme, Nanjing, China). Quantitative reverse transcription (qRT)-PCR was carried out in a total volume of 20 μL containing 2 μL of cDNA, 0.4 μL of gene-specific primers (10 μm), 10 μL of SYBR Green Mix, and 7.2 μL of RNase free ddH_2_O, using the Roche LightCycler 480 Real-time System (Roche, Basel, Swiss). The wheat *tubulin* gene was used as internal controls. The program and data analysis were carried out as described in the method suggested by Wang et al. (2018). Primers used for the qRT-PCR are designed by Primer5 listed in Supplementary Table 1. Three biological replications were performed. We obtained the *in silico* expression data of EXO70E1-V ortholog genes in wheat (*TraesCS3A02G302600*, *TraesCS3B02G333800*, and *TraesCS3D02G299200*) induced by *Pm* from the Triticeae Multi-omics Center wheat gene expression website^2^.

### Vector Construction and Subcellular Localization of *EXO70E1-V*

The ORF of *EXO70E1-V* (without the stop codon) was amplified by using primers green fluorescent protein (GFP)-EXO70E1-V-F/GFP-EXO70E1-V-R and then inserted into pAN580 vector as C-terminal fusions to the GFP reporter gene driven by the double 35S promoter. The GFP-EXO70E1-V vector was transformed into Yangmai158 protoplasts. Plasmid DNA (1.5 μg/μL) for each construct was mixed with a red fluorescent protein (RFP)/mCherry-fused marker protein (1.5 μg/μL), and 20 μL of total DNA was used to transform 200 μL of protoplasts derived from 5- to 7-day-old plants. Vectors expressing the PM marker PIP2a-mCherry and the trans-Golgi network (TGN)/early endosome marker mCherry-SYP61 were provided by Yiqun Bao (College of Life Science, Nanjing Agricultural University, Nanjing, China), and vectors for the endoplasmic reticulum (ER) marker (RFP-ER) and the Golgi marker (GmMan49–RFP) were provided by Libo Shan (Department of Plant Pathology and Microbiology, Texas A&M University, TX, United States). The GFP/RFP/mCherry signals were assessed by confocal imaging, 16–20 h after transformation. For imaging, an LSM780 confocal microscope (Zeiss, Jena, Germany)^3^ was used as described by Zhu et al. (2015). The primers are listed in Supplementary Table 1.

### Single-Cell Transient Overexpression Assay

*EXO70E1-V* was cloned into plant expression vector *pBI220* to generate vectors pBI220-EXO70E1-V (Supplementary Table 1). Transient overexpression assay (TOA) was performed according to Shirasu et al. (1999) and Cao et al. (2011). The reporter plasmid *pWMB002* containing the *β-glucuronidase* (*GUS*) gene and the expression plasmid were mixed before particle coating (molar ratio of 1:1; 1 μg of total DNA). The bombarded leaves were transferred to 1% agar plates supplemented with 85 μm benzimidazoles and incubated at 18°C for 8 h before inoculation of high density *Bgt* conidia spores. Leaves were stained at 48 hours after innoculation for identifying *GUS*-expressing cells, which is the indicator of cells transformed with *EXO70E1-V*. The average haustorium index (HI, percentage of *GUS*-stained cells with haustorium in the total *GUS*-staining cells attacked by *Bgt*) was computed based on the means of three independent experiments, each based on at least 100 independent interaction events.

### Wheat Transformation

The *EXO70E1-V* was overexpressed (driven by the CAMV 35S promoter) in Yangmai158 by stable transformation using the bombardment method according to Xing et al. (2008). The pAHC25 containing the reporter gene *GUS* and the herbicide tolerance gene driven by the *Ubi* promoter was used as a selectable vector. The pBI220-EXO70E1-V and pAHC25 vectors were co-transformed into calli cultured from immature embryos of wheat variety Yangmai158 by particle bombardment. Regenerated plants were produced as previously described (Xing et al., 2008). For screening of the positive transgenic plants, a specific primer pair (OE-EXO70E1-V-F/R, the product was 304 bp), which amplifies the sequence cover in the CaMV 35S promoter and part of *EXO70E1-V*, was designed to detect the transgene in plants from the T_0_ and T_1_ generations. Then, a pair of specific primers (T-Q-EXOE1-F and T-Q-EXOE1-R) that only detect the expression of *EXO70-V* from *H. villosa* were designed to detect the expression of *EXO70-V* in transgenic plants. The primers are listed in Supplementary Table 1.

## Results

### EXO70E1-V Interacted With CMPG1-V *in vivo* and *in vitro*

To elucidate the resistance pathway mediated by CMPG1-V, a Y2H cDNA library of *H. villosa* was constructed and used for screening using CMPG1-V as bait (Faheem et al., 2016). One positive fragment clone contains the typical Pfam03081 domain, which is typical for EXO70 superfamily. Based on the homologous cloning, the 1,815 bp full length of the gene was isolated from *H. villosa*, named EXO70E1-V, by the phylogenetic analysis with *Arabidopsis thaliana* (Supplementary Figure 1). EXO70E1-V protein is comprised of 605 amino acid proteins with the predicted molecular weight of 68.93 kDa and an isoelectric point of 5.04.

**FIGURE 1:**
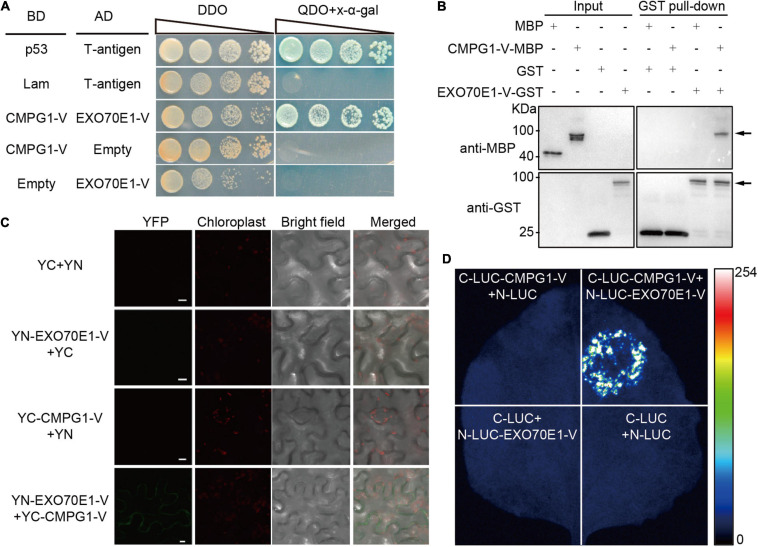
Interaction analysis between EXO70E1-V and CMPG1-V. **(A)** Analysis of EXO70E1-V and CMPG1-V interaction on double dropout or quadruple dropout medium by yeast two-hybrid (Y2H). Interaction of CMPG1-V (fused to BD, pGBKT7) with EXO70E1-V (fused to AD, pGADT7) by a Y2H experiment. p53 (pGBKT7-53)/T-antigen (pGADT7-T) and Lam (pGBKT7-Lam)/T-antigen were positive and negative controls, respectively. **(B)** *In vitro* EXO70E1-V/CMPG1-V interaction analysis using the GST pull-down assay. Anti-MBP antibody was used to detect the GST pull-down products, and the CMPG1-V-MBP could be detected only when co-incubated with EXO70E1-V-GST. Arrowheads indicate the CMPG1-V-MBP (upper panel) and the EXO70E1-V-GST (lower panel) proteins, respectively. **(C)** *In vivo* EXO70E1-V/CMPG1-V interaction analysis by BiFC assay in the *Nicotiana benthamiana* epidermal cells. The green channel shows the localization of the yellow fluorescent protein (YFP) and its fusion proteins, and the red channel shows the location of the chloroplast. YN and YC indicate N-terminal and C-terminal of YFP, respectively. Scale bar = 10 μm. **(D)** *In vivo* EXO70E1-V/CMPG1-V interaction analysis by LUI assay in the *N. benthamiana* epidermal cells. LUI image of *N. benthamiana* leaves co-infiltrated with the agrobacterial strains containing N-LUC-EXO70E1-V and C-LUC-CMPG1-V.

The interaction of full-length EXO70E1-V and CMPG1-V were verified by Y2H (Figure 1A). To test the direct interaction between EXO70E1-V and CMPG1-V, a GST pull-down assay was performed. The expected about 100 kDa fusion protein could be visualized only when GST-EXO70E1-V and MBP-CMPG1-V were co-incubated but not MBP (Figure 1B).

To confirm the interaction between EXO70E1-V and CMPG1-V *in vivo*, the BiFC and firefly LCI assays were operated. The results showed that only the co-expression of YN-EXO70E1-V and YC-CMPG1-V in tobacco leaves resulted in the complementation of fluorescence localized on the PM (Figure 1C). Meanwhile, the co-expression of CLUC-CMPG1-V and NLUC-EXO70E1-V in tobacco leaves could reconstitute a high luciferase activity, compared with the various negative controls (Figure 1D), and these results demonstrated that EXO70E1-V was able to interact with CMPG1-V both *in vitro* and *in vivo* (Figure 1).

### The *EXO70E1s* Were Conversely Present on the Homologous Group 3

**FIGURE 2:**
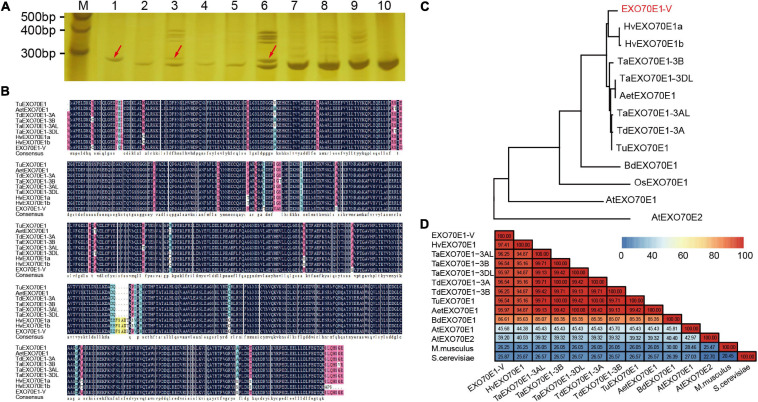
The sequence analysis of EXO70E1-V. **(A)** Chromosomal location of *EXO70E1-V* using a set of wheat-*Haynaldia villosa* addition lines. M, 20 bp DNA Ladder; 1, *H*. *villosa*; 2, Chinese Spring (CS); 3, *Triticum durum*–*H*. *villosa* amphiploid (AABBVV); 4–10, DA1V–DA7V. The red arrow indicates the target band. **(B)** Alignment of *EXO70E1* ortholog proteins in different *Triticeae* species. **(C)** Phylogenetic analysis of EXO70E1-V and its orthologs in *Triticeae* species, *Brachypodium distachyon*, *Oryza sativa*, and *Arabidopsis thaliana*. The *EX070* gene from *H. villosa* is marked in red. **(D)** Sequence similarity analysis of EXO70E1 ortholog proteins in *Triticeae* species, *A. thaliana*, *M. musculus*, and *S. cerevisiae*. Tu, *Triticum urartu*; Aet, *Aegilops tauschii*; Td, *Triticum dicoccoides*; Ta, *Triticum aestivum*; Hv, *Hordeum vulgare*; –V, *Haynaldia villosa*; *Os, Oryza sativa; Bd, Brachypodium distachyon.*

The chromosomal location of *EXO70E1-V* was determined by amplification using DNA from *Triticum durum–H. villosa* amphiploidy (genome AABBVV) and a complete set of Chinese Spring-*H. villosa* alien addition lines (DA1V–DA7V). A 285 bp product (YST-EXO70E1-V-F/R) was amplified in *H. villosa*, the amphiploid, and the addition line DA3V, but not in Chinese Spring, and the remaining tested addition lines. Thus, the *EXO70E1-V* was mapped to the chromosome 3V of *H. villosa* (Figure 2A).

In addition, we obtained the EXO70E1 protein orthologs from five Triticeae species (*T. aestivum, Triticum urartu, Aegilops tauschii, Triticum dicoccoides*, and *Hordeum vulgare*) by using the bioinformatics analysis. The EXO70E1 is a single copy in all five species and in all locations in homologous group 3 chromosomes. The alignment of *EXO70E1-V* with its orthologs indicated that EXO70E1s are well-conserved (Figure 2B). The results of the phylogenetic analysis showed obviously the branching between *Arabidopsis* and gramineous crops, and *EXO70E1-V* was closest to EXO70E1 from *H. vulgare* (Figure 2C). Through the sequence similarity analysis, the similarity of EXO70E1 orthologous genes was more than 94%, and the similarity between *EXO70E1-V* and *HvEXO70E1* is 97.41% (Figure 2D), which is consistent with the results of the phylogenetic analysis.

### The Subcellular Localization of EXO70E1-V

**FIGURE 3:**
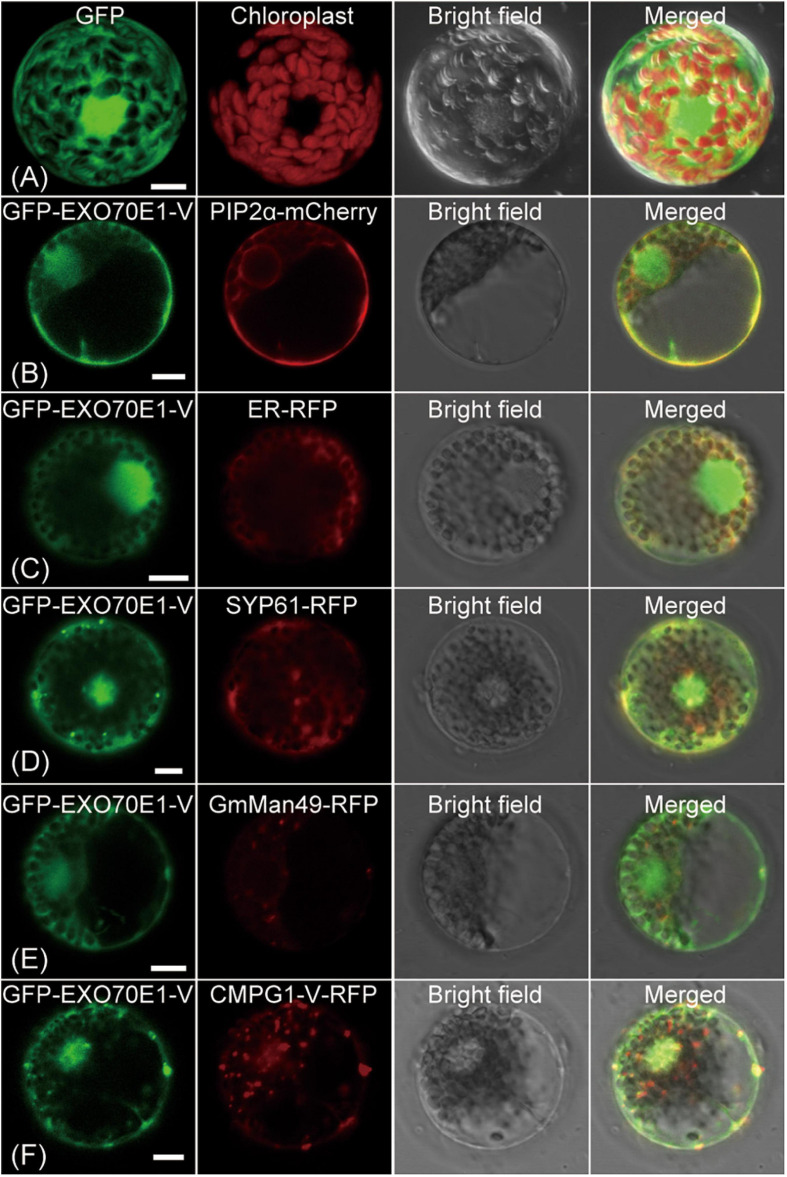
Subcellular localization of GFP-EXO70E1-V and interaction of GFP-EXO70E1-V with CMPG1-V-YFP in wheat protoplast. **(A)** GFP control. **(B)** Co-localization of the constructs GFP-EXO70E1-V (in green) and PIP2α–mCherry (in red, marker gene on plasma membrane). **(C)** Co-localization of the constructs GFP-EXO70E1-V (in green) and ER-RFP (in red, marker gene on endoplasmic reticulum). **(D)** Co-localization of the constructs GFP-EXO70E1-V (in green) and SYP61-RFP (in green, marker gene on trans-Golgi network/early endosome). **(E)** Co-localization of the constructs GFP-EXO70E1-V (in green) and GmMan49-RFP (in green, marker gene on Golgi). **(F)** Co-localization of GFP-EXO70E1-V (in green) and CMPG1-V-RFP (in red). Scale bar = 10 μm.

The biological function of protein is closely related to its subcellular location. To determine the subcellular localization of EXO70E1-V, a co-expression vector containing EXO70E1-V and GFP-EXO70E1-V was constructed and transformed into wheat protoplasts. Compared with the ubiquity of GFP signals in the cell (Figure 3A), GFP-EXO70E1-V signals were mainly distributed in the nucleus and on the PM and also in some cytoplasmic signals (Figure 3B). To determine the specific location, the marker genes for PM (*PIP2a-mCherry*), ER (*RFP-ER*), TGN/early endosome vesicle *(RFP-SYP61*), and Golgi (*GmMan49-RFP*) were co-transformed with GFP-EXO70E1-V, and the GFP signal was found to overlap with PM, ER, and TGN but not Golgi (Figures 3B–E). These results indicate that EXO70E1-V is located in the nucleus, PM, ER, and TGN. When GFP-EXO70E1-V and RFP-CMPG1-V were co-transformed into wheat protoplasts, dot-like structure fluorescence signals were partly colocated in PM and nucleus (Figure 3F). This result indicated the possibility of participation of EXO70E1-V and CMPG1-V together in similar biological processes.

### Expression Profiling of *EXO70E1s*

**FIGURE 4:**
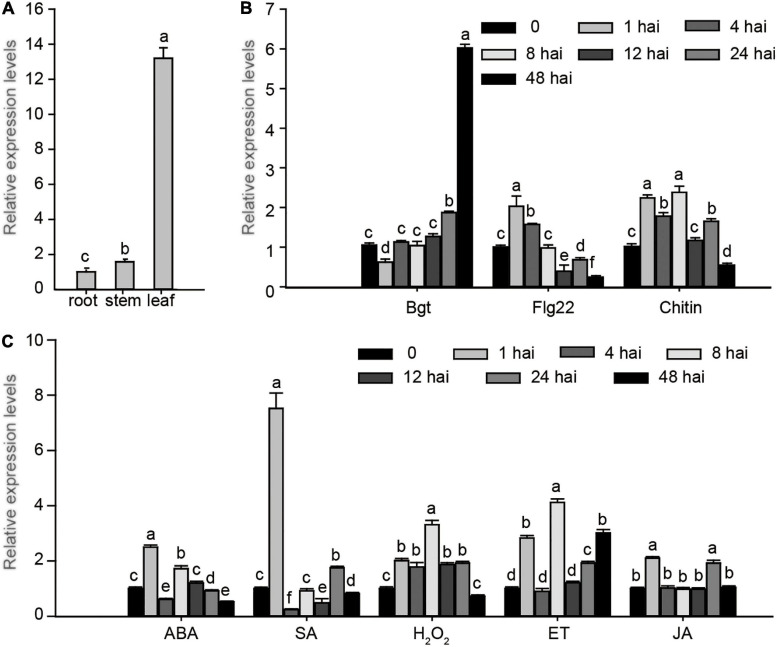
Expression profiling of *EXO70E1-V.* **(A)** The expression of *EXO70E1-V* in root, stem, and leaf of *H*. *villosa*. **(B)** Expression levels of *EXO70E1-V* in biotic stresses of *H. villosa*. **(C)** Expression profiling of *EXO70E1-V* under stress-related signal treatments. *Bgt, Blumeria graminis* f.sp *tritici*; SA, salicylic acid; MeJA, methyl jasmonate; ET, ethephon; ABA, abscisic acid; H_2_O_2_, hydrogen peroxide. Each column represents the means ± SD. Different lowercase letters represent significant differences at 5% level according to the least significant difference (LSD) test.

To explore the potential biological function of *EXO70E1-V* genes in different organs and in response to stress treatments, the qRT-PCR was performed. The expression of *EXO70E1-V* showed a high expression level in leaves than in stems and roots (Figure 4A). *EXO70E1-V* was upregulated and reached a peak at 48 h after *Bgt* inoculation, which was about 6-fold compared with non-inoculated *H. villosa*, but hardly induced by flg22 and chitin (Figure 4B). *EXO70E1-V* was quickly induced by SA, which showed the tendency of quickly increasing first and then decreasing rapidly, got the peak value after 1 h treatment, that was about 7.5-fold compared with control and then back to a relatively lower level (Figure 4C). The *in silico* expression analysis of *EXO70E1-V* ortholog genes (*TraesCS3A02G302600*, *TraesCS3B02G333800*, and *TraesCS3D02G299200*) in common wheat (Chinese Spring, susceptible) showed that they were not induced by *Pm* (Supplementary Figure 2).

### Functional Analysis of *EXO70E1-V* in *Pm* Resistance

**FIGURE 5:**
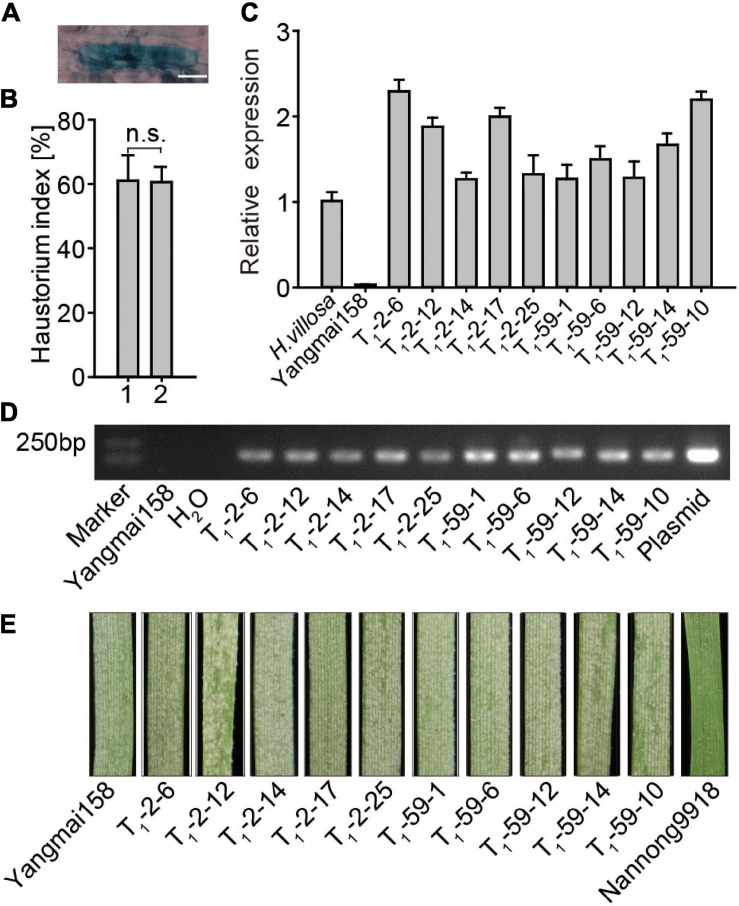
Functional analysis of *EXO70E1-V* in powdery mildew (*Pm*) resistance. **(A)** Examples of *β-glucuronidase* (*GUS*) staining and haustorium, scale bar = 20 μm. **(B)** Transient overexpression assay (TOA) of *EXO70E1-V*. 1 and 2 represent vectors *GUS* alone and *GUS* + EXO70E1-V, respectively. Haustorium index is the percentage of *GUS*-stained cells having haustorium in the total *GUS*-staining cells attacked by *Bgt*. Each column represents the means ± SD. n.s. represents not significant. **(C)** *EXO70E1-V* expression in *H. villosa*, receptor Yangmai158, and two T_1_ transgenic lines (T_1_-2 and T_1_-59). The expression levels were normalized to *Tubulin*, and *H. villosa* is set as “1.” **(D)** Amplification for *EXO70E1-V* in T_1_ transgenic lines. Yangmai158 and H_2_O were negative controls, and plasmid pBI220-EXO70E1-V was used as a positive control. Marker: DL2000 DNA ladder. **(E)** Observation of *Pm* spores on leaves. Yangmai158 and Nannong9918 were used as susceptible and resistant control, respectively.

The function of *EXO70E1-V* in *Pm* resistance was validated using single cell TOA and stable transformation. The formation of haustorium indicates successful penetration of *Bgt* into wheat epidermal cells (Figure 5A). A higher HI indicates increased *Pm* susceptibility. For TOA, *GUS* alone and *GUS* + *EXO70E1-V* constructs were bombarded into leaves of a moderate *Pm* susceptible wheat variety Yangmai158. The HI values in the *GUS-*expressing epidermal cells were compared. The average HI in the *GUS-*expressing epidermal cells showed no significant difference in plants transforming *GUS* alone (HI: 60.97%) and plants co-transforming *GUS* + *EXO70E1-V* (HI: 60.54%) (Figure 5B). This indicated that transient overexpression of *EXO70E1-V* in Yangmai158 could not change its HI.

The regeneration plant was obtained by co-transforming *pBI220-EXO70E1-V* and *pAHC25* (carrying bar gene as a selection marker) vectors into 1,000 young embryo calluses of Yangmai158 by using the particle bombardment-mediated transformation approach. The T_1_ lines generated from EXO70E1-V-T_0_-2 and EXO70E1-V-T_0_-59 were selected for further analysis. The result of qRT-PCR showed that the expression level of *EXO70E1-V* varied in the T_1_ transgenic plants and was comparable with *H. villosa* (contains *EXO70E1-V*), but no expression of *EXO70E1-V* was detected in the Yangmai158 (does not contain *EXO70E1-V*) (Figure 5C). The agarose gel electrophoresis analysis showed that EXO70E1-V existed in each of these two transgenic lines (Figure 5D). However, T_1_ lines were showed no obvious effect on *Pm* resistance at the seedling when detached leaves were inoculated with a mixture of *Bgt* isolates (Figure 5E). The results further proved that the overexpression of *EXO70E1-V* in Yangmai158 could not change the resistance to mixed *Bgt.*

## Discussion

EXO70 acts as a key member of the exocyst complex, formed by three distinct subfamilies (EXO70.1, EXO70.2, and EXO70.3) (Elias, 2003), which functions in cell and organ morphogenesis, stress responses, and hormone signaling (Synek et al., 2006; Zhao et al., 2015; Sabol et al., 2017; Guo et al., 2018). In this study, we identified an EXO70 isoform by Y2H screening and cloned from *H. villosa*, named EXO70E1-V. The results of the experiment showed that EXO70E1-V was able to interact with wheat *Pm* positive regulator CMPG1-V both *in vitro* and *in vivo* (Figure 1), and the transcript could be rapidly induced by *Pm* and exogenous SA (Figure 4), speculating that *EXO70E1-V* might play a role in wheat *Pm* resistance.

Subcellular localization is a key characteristic of protein functional research. In previous studies, AtEXO70E1 has mainly distributed a diffuse cytoplasmic signal in the protoplasts of *A. thaliana* (Ding et al., 2014). In this study, the GFP-EXO70E1-V fusion protein was transformed into wheat protoplasts, and the result revealed its main localization in the PM and nucleus. The incomplete consistent result possibly explained by different species has different evolutionary environments, and to better adapt to the change of the environment, the gene may evolve a new function based on the original function (Roulin et al., 2013). In addition, the co-localization fluorescence signals of GFP-EXO70E1-V and RFP-CMPG1-V proteins were mainly in PM and nucleus with the dot-like structure. There are a few reports about the ubiquitination of PM proteins in plants, including auxin efflux carrier PIN2, water channel aquaporin PIP2, flagellin receptor FLS2, and iron transporter IRT1 (Abas et al., 2006; Lee et al., 2009; Barberon et al., 2011; Lu et al., 2011). This result indicated the possibility of EXO70E1-V and CMPG1-V participation in some overlapping biological process, and the location of the PM may be related to its function. CMPG1-V is localized in the PM, nucleus, ER, and partially in TGN/early endosome vesicles (Zhu et al., 2015). Combined with the result of BiFC that showed fluorescence signal in PM, we speculated that after EXO70E1-V interacted with CMPG1-V, they mainly function on PM. When the *Bgt* infected, they enriched on PM to identify the infection of *Bgt* whether EXO70E1-V is modified by CMPG1-V ubiquitination on the PM and how its functions can be further studied.

The study showed that the members of EXO70A, EXO70C, and EXO70G isoforms are usually linked with the growth and development of plants (Lai, 2016; Synek et al., 2017; Du et al., 2018). *Arabidopsis* EXO70A1, the best characterized members of EXO70A isoform, involved in cytokinesis (Fendrych et al., 2010), root hair and cell growth (Synek et al., 2006; Wu et al., 2013; Cole et al., 2014), pollen–stigma interactions (Safavian et al., 2015), hypocotyl development (Hala et al., 2008; Jankova Drdova et al., 2019), and primary and secondary cell wall biogenesis (Li et al., 2013; Oda et al., 2015; Vukasinovic et al., 2017). Instead, EXO70B, E, F, and H isoforms are often related to plant biotic interactions and defense responses. Among them, two members of *Arabidopsis* EXO70B clade (i.e., EXO70B1 and EXO70B2) are the best representatives, which play an important role in autophagy-related transport, stomatal regulation, and plant immunity (Pecenkova et al., 2011; Stegmann et al., 2012; Kulich et al., 2013; Hong et al., 2016; Seo et al., 2016).

In our previous study, the phylogenetic analysis revealed that EXO70E1-V, together with AtEXO70E1/E2 and OsEXO70E1 fell into the E clade of EXO70 proteins (Zhao et al., 2018). AtEXO70E2 can recruit other subunits to form a new organelle function on an unconventional secretory pathway, but no functional studies have been reported so far, so does its closest homolog AtEXO70E1 (Wang et al., 2010; Ding et al., 2014; Lin et al., 2015). OsEXO70E1 is involved in defense against herbivorous planthoppers by interacting with Bph6, which increases the exocytosis and leads to cell wall thickening (Guo et al., 2018). In this study, transient and stable overexpression of *EXO70E1-V* had no obvious effect on the resistance of wheat when compared with the Yangmai158 (Figure 5). Consequently, we speculated that the function of EXO70E1-V was possibly more inclined to AtEXO70E2, which is only responsible for recruiting other subunits to the area of active secretion, rather than participating in the later biochemical process. The overexpression of *EXO70E1-V* does not increase the resistance of wheat to *Pm*, which indicated that it may require the participation of other proteins, such as its interaction protein CMPG1-V. Meanwhile, we also transiently silenced *EXO70E1-V* gene in *H. villosa* by virus-induced gene silencing using the barley stripe mosaic virus-mediated system; however, we did not change the *Pm* resistance level of *H. villosa* (data not shown). We suspected that it may be due to the presence of *Pm21* that showed high resistance to *Pm* (Cao et al., 2011). The function of EXO70E1-V in *Pm* resistance needs to be further verified.

The phytohormone SA is closely related to disease resistance by inducing the expression of disease-related (PR) genes or as an early signal component (Vlot et al., 2009). H_2_O_2_ accumulates in plant mesophyll cells, it induces the hypersensitive reaction, and the oxidative burst (production of ROS, including H_2_O_2_) was related to the resistance of plants to the pathogen. In a previous study, the expression of *CMPG1-V* in *H. villosa* was increased when treated with SA, ABA, and H_2_O_2_, and the CMPG1-V transgenic plants show improved *Pm* resistance due to enhanced expression of SA-responsive genes and H_2_O_2_ accumulation (Zhu et al., 2015). Our results showed that *EXO70E1-V* was also quickly induced by SA treatments, and EXO70E1-V and CMPG1-V were interacting on the PM. This indicates that CMPG1-V and EXO70E1-V may participate in the same hormone pathway. We speculated that EXO70E1-V participates in the transmission of different hormone signals in the process of CMPG1-V disease resistance to help CMPG1-V resist the infection of *Pm*.

## Data Availability Statement

The raw data supporting the conclusions of this article will be made available by the authors, without undue reservation, to any qualified researcher.

## Author Contributions

JX, XW, JZ, and HZ conceived and designed the study and wrote the manuscript. JZ, HZ, XZ, ZW, YN, and YC analyzed the data. JZ, LS, HW, and HZ collected the plant materials. JZ, HZ, and XZ performed the experiments. All authors reviewed and edited the manuscript.

## Conflict of Interest

The authors declare that the research was conducted in the absence of any commercial or financial relationships that could be construed as a potential conflict of interest.

## Footnotes

1 http://plants.ensembl.org/index.html

2 http://202.194.139.32/expression/index.html

3 http://www.zeiss.com
