# Supplementary material for: The Exocyst Complex Subunit EXO70E1-V From Haynaldia villosa Interacts With Wheat Powdery Mildew Resistance Gene CMPG1-V
Source: Front Plant Sci. 2021 Jul 8;12:652337. doi: 10.3389/fpls.2021.652337 (PMC8295898; doi:10.3389/fpls.2021.652337)
Supplement: Supplementary file 1 [file Table_1.docx]

Supplementary Material

**Table S1** The primers used in this study.

| **Primer name** | **Primer sequences** | **Used for** |
| --- | --- | --- |
| AD-EXO70E1-V-F | CCATGGAGGCCAGTGAATTCATGATGGCTCCTGAGTTAG | Construct pGADT7- EXO70E1-V |
| AD-EXO70E1-V-R | ACGATTCATCTGCACTCGAGTTCAGCTCGGCCAGAATGCTG |  |
| BD-CMPG1-V-F | GCCATGGAGGCCGAATTCATGGTCACGCCGCTGCCCC | Construct pGBKT7- CMPG1-V |
| BD-CMPG1-V-R | AGCTCGAGCTCGATGGATCCTCAGAAGGGTCTCTTGAGC |  |
| nLUC- EXO70E1-V-F | GGGGTACCATGATGGCTCCTGAGTTAG | Construct NLuc-EXO70E1-V |
| nLUC- EXO70E1-V-R | GCGTCGACAGCTCGGCCAGAATGCTGC |  |
| YST-EXO70E1-V-F | GCATCCCCTCACCAAGTATG | For chromosomal location |
| YST-EXO70E1-V-R | TCCGAGTTCTTCACTTTCTGG |  |
| cLUC-CMPG1-V-F | CGGTACCCGGGATCCATGGTCACGCCGC | Construct cLuc-CMPG1-V |
| cLUC-CMPG1-V-R | TGAGACCAGTTAATTAAGAAGGGTCTCTTGA |  |
| YC-CMPG1-V-F | CCTTAATTAACATGGTCACGCCGCTGCCCCGCT | Construct YC-CMPG1-V |
| YC-CMPG1-V-R | GGACTAGTGAAGGGTCTCTTGAGCGCCTT |  |
| YN- EXO70E1-V-F | CCTTAATTAACATGATGGCTCCTGAGTTAGA | Construct YN-EXO70E1-V |
| YN- EXO70E1-V-R | GGACTAGTAGCTCGGCCAGAATGCTGCAA |  |
| GST- EXO70E1-V-F | GCGTCGACATGATGGCTCCTGAGTTAG | Construct GST-EXO70E1-V |
| GST- EXO70E1-V-R | CCTCGAGTCAAGCTCGGCCAGAATGC |  |
| EXO70E1-V-Q-F | CATCGTGGAGTCAGGTGC | qRT-PCR for expression profiling of *EXO70E1-V* |
| EXO70E1-V-Q-R | CCATCAAGGAAGGAGGAG |  |
| pAN580-EXO70E1-V-F | GGACTAGTATGATGGCTCCTGAG | Sub-cellular localization for *EXO70E1-V* |
| pAN580-EXO70E1-V-R | TCCCCCGGGAGCTCGGCCAGAATGCT |  |
| pBI220-EXO70E1-V-F | TGGAGAGAACACGGGGGATCCATGATGGCTCCTGAGTTAG | Over-expression vector Construction for *EXO70E1-V* |
| pBI220-EXO70E1-V-R | AACGTCGTATGGGTAAGGCCTAGCTCGGCCAGAATGCTGC |  |
| OE- EXO70E1-V-F | CGCACAATCCCACTATCC | Identification positive transgenic plants |
| OE- EXO70E1-V-R | TCTCCACCAGGGTCCAA |  |
| T-Q-EXOE1-F | GGATTTCTCTGCTGACAT | qRT-PCR for transgenic expression profiling of *EXO70E1-V* |
| T-Q-EXOE1-R | AAGCACCTGACTCCACGAT |  |
| *Tubulin*-F | GATGCAGCCAACAACTTCGCC | qRT-PCR for expression of *Tubulin* |
| *Tubulin*-R | CAGTTCCACCTCCAACAGCGT |  |
