# Supplementary material for: The Exocyst Complex Subunit EXO70E1-V From Haynaldia villosa Interacts With Wheat Powdery Mildew Resistance Gene CMPG1-V
Source: Front Plant Sci. 2021 Jul 8;12:652337. doi: 10.3389/fpls.2021.652337 (PMC8295898; doi:10.3389/fpls.2021.652337)
Supplement: Supplementary file 2 [file Data_Sheet_1.docx]

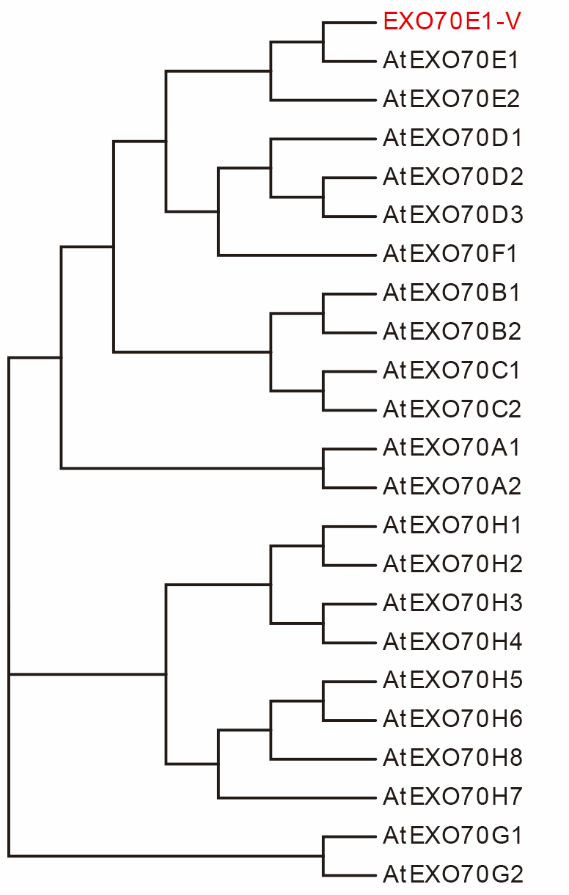
Supplementary Material

**Figure S1** Phylogenetic analysis of EXO70E1-V and its ortholog proteins in *Arabidopsis thaliana*. The EXO70E1-V was red.
