# Supplementary material for: The Exocyst Complex Subunit EXO70E1-V From Haynaldia villosa Interacts With Wheat Powdery Mildew Resistance Gene CMPG1-V
Source: Front Plant Sci. 2021 Jul 8;12:652337. doi: 10.3389/fpls.2021.652337 (PMC8295898; doi:10.3389/fpls.2021.652337)
Supplement: Supplementary file 3 [file Data_Sheet_2.docx]

Supplementary Material


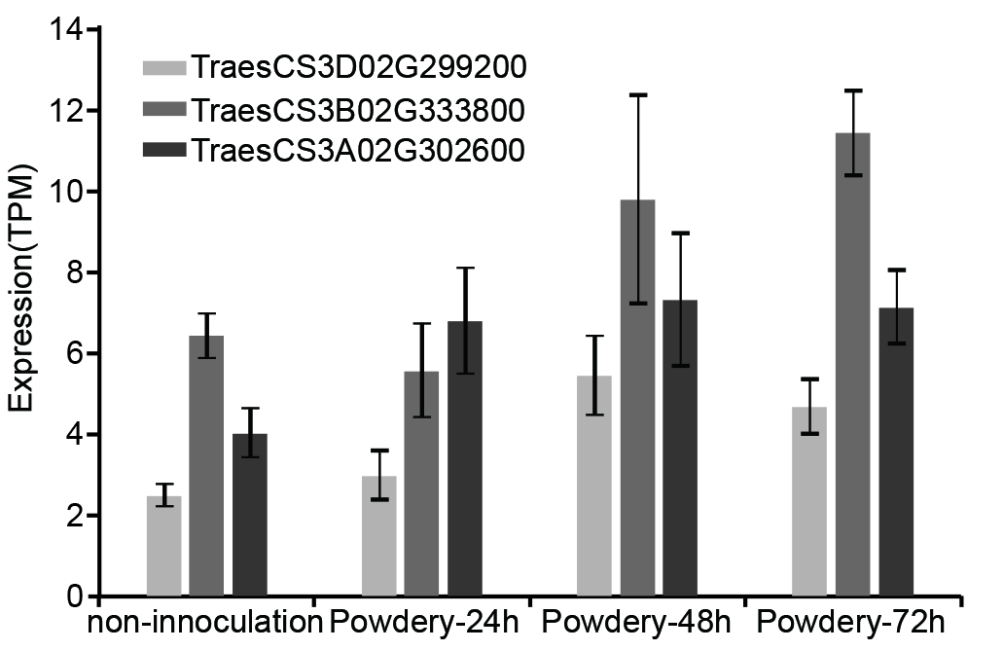


**Figure S2** *In silico* expression profiling analysis of *EXO70E1-V* orthologs in wheat induced by powdery mildew*.* The expression data of *EXO70E1-V* ortholog genes in wheat (*TraesCS3A02G302600*, *TraesCS3B02G333800*, *TraesCS3D02G299200*) induced by powdery mildew were download from the Triticeae Multi-omics Center wheat gene expression website (http://202.194.139.32/expression/index.html). Each column represents the average value of expression at each time point.
